# The Impact of Substance Abuse Problems and Serious Mental Illness/Serious Emotional Distress on Post‐Discharge Residential Status Among Clients With Behavioral and Cognitive Disorders: Evidence From SAMHSA MH‐CLD Data

**DOI:** 10.1002/puh2.70218

**Published:** 2026-04-10

**Authors:** Eden Moges, Norma Rochez, William Peycha, Aditya Chakraborty

**Affiliations:** ^1^ Macon and Joan Brock Virginia Health Sciences Old Dominion University Norfolk Virginia USA

**Keywords:** behavioral and cognitive disorders, housing insecurity, integrated care, social determinants of health, substance abuse problems

## Abstract

**Background:**

Behavioral and cognitive disorders can undermine housing stability, a key determinant of mental health recovery, with persistent disparities across demographic and socioeconomic groups. This study examined the associations of substance abuse problems (SAP) and serious mental illness/serious emotional distress (SMISED) with residential status at discharge from mental health facilities.

**Methods:**

This cross‐sectional study used the utilized data from the Substance Abuse and Mental Health Services Administration (SAMHSA). Descriptive statistics were used to summarize demographic characteristics, whereas univariate and multivariable logistic regression models were employed to assess associations between the covariates and the residential outcome, adjusting for a variety of demographic characteristics.

**Results:**

In the adjusted multivariable model, SAP was linked to 40% increased odds of non‐private discharge (adjusted odds ratio [aOR] = 1.40, 95% confidence interval [CI]: 1.18–165), and SMISED had about two‐fold increased odds (aOR = 2.10, 95% CI: 1.44–3.05) compared to discharge in private residence. For individuals with SAP, unemployed Hispanic/Latino clients showed the highest odds of non‐private discharge compared to employed clients (aOR = 3.74, 95% CI: 1.65–8.46). For individuals with SMISED, unemployed Black clients had approximately four‐fold higher odds (aOR = 4.02, 95% CI: 2.00–8.37) of non‐private discharge, compared with employed clients.

**Conclusion:**

These findings suggest that housing stability after discharge reflects the combined effects of clinical severity and socioeconomic disadvantage, with higher risk associated with substance use, unemployment, and racial inequities. Integrated mental health and housing interventions, including culturally responsive policies, are needed to improve residential stability among vulnerable populations.

## Graphical abstract

SAP was linked to 40% increased odds of non‐private discharge (aOR = 1.40, 95% CI: 1.18–165), and SMISED had about two‐fold increased odds (aOR = 2.10, 95% CI: 1.44–3.05) compared to discharge in private residence. For individuals with SAP, unemployed Hispanic/Latino clients showed the highest odds of non‐private discharge compared to employed clients (aOR = 3.74, 95% CI: 1.65–8.46). For individuals with SMISED, unemployed Black clients had approximately four‐fold higher odds (aOR = 4.02, 95% CI: 2.00–8.37) of non‐private discharge, compared with employed clients.

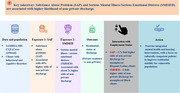

## 1 Introduction

Mental health is a complex and multifactorial concept influenced by a wide range of biological, psychological, and social factors. Among these, behavioral and cognitive disorders, including Attention‐Deficit/Hyperactivity Disorder (ADHD), Oppositional Defiant Disorder (ODD), Conduct Disorder, Personality Disorders, and Pervasive Developmental Disorder (PDD), represent a group of conditions that substantially affect functioning across social, academic, and occupational domains [16]. Although these disorders often manifest during childhood and adolescence, their effects can persist in adulthood, influencing critical life transitions such as employment stability, social integration, and independent living. Individuals with these disorders frequently experience challenges in emotion regulation, executive functioning, and social interaction, which can contribute to the difficulties navigating essential systems. These vulnerabilities become especially pronounced during transitions from mental health facilities back to community settings, where residential stability is closely tied to long‐term recovery and well‐being [7].

Stable housing is a foundational determinant of health and well‐being, particularly for individuals with mental and behavioral health conditions [13]. For individuals with behavioral disorders, maintaining stable housing is often compromised by difficulties managing financial responsibilities, sustaining relationships with caregivers or landlords, and engaging consistently with treatment and social service systems. Prior research has consistently demonstrated that individuals with behavioral and cognitive disorders are disproportionately represented among populations experiencing homelessness, residential instability, and placement in supervised or institutional living arrangements [7, 14]. These patterns reflect not only individual‐level impairments but also broader systemic gaps in housing availability, service coordination, and long‐term community‐based support.

The transition from mental health facilities to community settings represents a particularly vulnerable period for individuals with behavioral disorders. Discharge marks a shift from structured, supervised care to reliance on external systems, including family networks, housing markets, and community mental health services. Youth and adults exiting inpatient or residential treatment settings often do so in the context of limited housing options, fragmented discharge planning, and insufficient access to follow‐up services [4]. Without adequate supports, individuals may be discharged into unstable or restrictive living environments, including homelessness, temporary housing, group homes, foster care placements, or correctional facilities. Consequently, residential status at discharge serves as a critical indicator of continuity of care, system capacity, and the broader social conditions shaping post‐treatment outcomes.

With an adapted social determinant of health (SDOH) framework and social‐ecological viewpoint, we conceptualize post‐discharge residential status as the outcome of multilevel factors, which are not limited to clinical symptoms alone. The SDOH framework emphasizes that health and social outcomes are not determined solely by clinical diagnosis but are profoundly shaped by the social, economic, and structural environments in which individuals live [13]. At the structural and policy level, the socioeconomic status of individuals, the affordability/availability of housing, and supportive services determine whether an individual is capable of obtaining and maintaining stable housing. These determinants at the upstream level influence the intermediate mechanisms of change, including material hardship, psychosocial stress, and later continuity of care. At the individual level, behavioral/cognitive disorders and serious mental illness or emotional distress (SMISED) can impair executive functioning, daily living skills, and relationship stability, whereas co‐occurring substance use may further disrupt employment, strain social supports, and complicate engagement with treatment, collectively increasing vulnerability to residential instability after discharge [18]. Such multilevel framing is aligned and consistent to those ecological models that focus on interactions between individual, interpersonal, social, and policy contexts in influencing health‐related outcomes [19, 20].

Notably, the shift of inpatient psychiatric care into the community is a risky phase in which housing pathways may quickly become unstable unless mental health service users are provided with coordinated supports; randomized evidence indicates that targeted transitional services (e.g., Critical Time Intervention [CTI]) can decrease the rate of future homelessness in the individuals with severe mental illness who have their psychiatric discharge [21].

Prior research shows how the impact of community‐level factors plays a central role in shaping discharge outcomes for individuals with behavioral disorders. Under‐resourced neighborhoods, shortages of behavioral health providers, limited availability of supportive housing, and weak coordination between inpatient facilities and community‐based services can constrain housing options at the point of discharge [14]. Families and caregivers are often expected to assume primary responsibility for post‐discharge care, despite experiencing caregiver burden, financial strain, or limited access to appropriate services. These challenges increase the likelihood that individuals with behavioral disorders transition from treatment settings into non‐private or unstable living arrangements.

In addition, structural inequities further compound these risks. Individuals with behavioral disorders are more likely to experience comorbid substance use, limited access to culturally competent care, and disproportionate involvement with child welfare or juvenile justice systems, all of which are strongly associated with housing instability [10, 17]. Racial and ethnic minorities face additional barriers related to systemic discrimination, stigma surrounding mental illness, and reduced access to mental health services. These further limit housing opportunities and undermine discharge planning efforts [5, 10], highlighting the need to examine residential outcomes through a structural lens that accounts for both social disadvantage and institutional barriers.

Recognizing the intersection between mental health and housing, global and national organizations have emphasized the importance of integrated and recovery‐oriented approaches to care. The World Health Organization [13] has called for systemic reforms that address housing as a core component of mental health care. Moreover, the Substance Abuse and Mental Health Services Administration (SAMHSA) emphasizes the use of client‐level data to evaluate outcomes related to services utilization and recovery as part of its National Outcomes Measures framework [11]. Despite this emphasis, gaps remain when understanding how behavioral disorders specifically influence residential status, at discharge from a facility, across diverse populations and service settings.

Overall, this study seeks to clarify how SMI identifies distress and substance abuse problems (SAP) operating within these multilevel determinants to shape post‐discharge residential outcomes among the individuals with behavioral/cognitive disorders and to identify patterns that reflect broader social and structural inequities. Findings will inform policy and practice efforts aimed at improving discharge planning, strengthening community support, and reducing housing instability among individuals with behavioral disorders. By understanding these associations, this study seeks to inform policies that integrate mental health care with housing support, ultimately aiming to reduce housing disparities for individuals with mental health conditions.

## 2 Methods

### 2.1 Study Design and Data Source

This study employed a cross‐sectional design using the Mental Health Client‐Level Data (MH‐CLD) [6], provided by the SAMHSA. The MH‐CLD dataset is a nationally representative database that includes comprehensive data on individuals receiving mental health services across State Mental Health Agencies (SMHAs) in the United States. It contains detailed information on participants’ demographics, clinical diagnoses, service utilization, and residential outcomes. The MH‐CLD dataset is weighted to account for complex survey design, nonresponse, and noncoverage, allowing estimates to generalize to the national population of individuals receiving public mental health services. The dataset remains the most recent nationally comprehensive client‐level surveillance system available with standardized diagnostic and residential outcome measures.

#### 2.1.1 Study Population

The initial MH‐CLD 2022 dataset included data on 1,048,575 participants. From the overall sample, we selected individuals with cognitive and behavioral disorders (*n* = 172,643). Our final sample included 18,211 participants after removing the missing observations from all variables. The study focused on participants with behavioral and cognitive disorders, and the association of some clinical/behavioral health indicators on residential status and other relevant covariates among the participants.

### 2.2 Measures

#### 2.2.1 Behavioral and Cognitive Disorders

Behavioral and cognitive disorders were operationalized as a composite indicator reflecting the presence of at least one of the following diagnoses: ADHD, ODD, Conduct Disorder, Personality Disorder, or PDD. Individuals with one or more of these diagnoses were categorized as having a behavioral or cognitive disorder and included in the study.

This composite approach aligns with prior population‐level studies using administrative mental health datasets, where diagnostic co‐occurrence is common, and diagnostic granularity varies across reporting agencies. The composite specification captures the functional burden associated with externalizing and neurodevelopmental disorders and reflects real‐world clinical complexity.

#### 2.2.2 Primary Exposure

- SAP: Specifies the client's substance use problem based on a substance use diagnosis and/or using other identification method such as substance use screening results, enrollment in a substance use program, substance use survey, service claims information, or other related sources of data. This variable was coded as “yes” and “no” based on whether an individual reported the substance abuse.
- SMISED: Categorized as SMI, SED, or at risk for SED, and no SMI/SED. The variable SMISED was dummy coded as “yes” by combining SMI and SED together and “no” by taking the category no SMI/SED.

#### 2.2.3 Outcome: Residential Status

The primary outcome variable, residential status, was classified into a binary variable:

- Private residence: Individuals discharged to a private residence, including family homes.
- Non‐private residence: Individuals discharged to homelessness or other institutional settings such as foster care, residential treatment facilities, or correctional institutions.

#### 2.2.4 Covariates

Several covariates were included to adjust for potential confounding factors:

1. Gender: Categorized as male or female.
2. Education level: Coded into three categories: Less than high school, special education, and high school attendees or graduate.
3. Employment status: Categorized as employed or unemployed/not reported. Race: Categorical variable including non‐Hispanic Black, non‐Hispanic White, and Others (American Indian/Alaska Native, Asian, Native Hawaiian or Other Pacific Islander).
4. Ethnicity: Categorical variable coded as Hispanic (Mexican, Puerto Rican, and other Hispanic or Latino origin) and non‐Hispanic (not of Hispanic or Latino origin).
5. Age group: Categorized as 0–17, 18–44, and 45+ years.
6. Number of mental health diagnoses reported (NUMMHS): The number of mental health diagnoses that were already reported for each client, which ranges from 0 to 3.
7. Marital status: Divided into four categories: married, unmarried, separated, and divorced.

### 2.3 Statistical Analysis

Descriptive statistics were used to summarize the demographic characteristics of the study sample and the prevalence of behavioral and cognitive disorders and residential status. To examine associations between the primary exposure, residential status, and covariates, chi‐square tests were conducted for categorical variables, whereas *t*‐tests were applied for continuous variables. Four logistic regression models were developed. The first model, an unadjusted logistic regression, assessed the crude relationship between the associated covariates and residential status. The second model, a multivariable logistic regression, adjusted for covariates, estimated the adjusted odds ratio (aOR) for the likelihood of being discharged to a non‐private residence compared to a private residence.

The third model was developed using the “backward stepwise elimination” approach to identify the most parsimonious model while minimizing issues related to multicollinearity. The fourth model evaluated the moderating effect of SAP and SMISED on the employment status for predicting the outcome of residential status. In the moderation analysis, the differences in likelihood of discharge to non‐private residence between unemployed (vs. employed) clients based on SAP and SMISED status were measured. The moderation was achieved by introducing an interaction term between the employment status and each moderator (employment status × SAP and employment status × SMISED) in the multivariate logistic regression models, adjusting for the covariates. The odds ratios (ORs) and aORs with 95% confidence intervals (CIs) were reported to quantify the strength and direction of associations. Statistical significance was set at *p* < 0.001 for all tests. All analyses were conducted using SAS software (Version 9.4).

## 3 Results

Table 1 presents a summary of 18,211 clients, 17,356 of which (95.3) were discharged to a private residence and 855 (4.7) to a non‐private residence. The study sample was well balanced across males (54.02%) and females (45.98%) and was predominantly non‐Hispanic White (58.24%), followed by non‐Hispanic Black (32.84%) and other non‐Hispanic racial groups (8.94%). The sample was predominantly non‐Hispanic, with 17,218 clients (94.54%), whereas 993 clients (5.45%) identified as Hispanic. The demographic breakdown shows a diverse population, enabling an analysis of how different racial and ethnic groups with specific behavioral and cognitive disorders are affected by the SAP and serious mental illness in terms of residential stability.

**TABLE 1:** Bivariate associations between participant characteristics and residential status at discharge.

Variables	Residential status		*p* value
	Private residence *N* (%)	Non‐private residence *N* (%)	
**Primary exposures**			
**Substance abuse problem (SAP)**			
No	14,740 (80.94)	618 (3.39)	<0.0001
Yes	2616 (14.36)	237 (1.30)	
**Serious mental illness and emotional distress (SMISED)**			
No	987 (5.42)	30 (0.16)	0.007
Yes	16,369 (89.89)	825 (4.53)	
**Covariates**			
**Sex**			
Male	9365 (51.42)	474 (2.60)	<0.0001
Female	7991 (43.88)	381 (2.09)	
**Age**			
0–17	9717 (53.36)	222 (1.22)	<0.0001
18–44	4700 (25.81)	342 (1.88)	
45+	2939 (16.14)	291 (1.60)	
**Race**			
Non‐Hispanic Black	5722 (31.42)	258 (1.42)	0.0006
Non‐Hispanic White	10,059 (55.24)	545 (2.99)	
Non‐Hispanic Other	1575 (8.65)	52 (0.29)	
**Ethnicity**			
Hispanic	958 (5.26)	35 (0.19)	0.073
Non‐Hispanic	16,398 (90.04)	820 (4.50)	
**Education**			
Less than high school	11,542 (63.38)	397 (2.18)	<0.0001
Special education	62 (0.34)	9 (0.05)	
High school attendees or graduate	5752 (31.59)	449 (2.47)	
**Employment status**			
Employed	1744 (9.58)	50 (0.27)	<0.0001
Unemployed	15,612 (85.73)	805 (4.42)	
**Marital status**			
Married	1774 (9.74)	67 (0.37)	<0.0001
Unmarried	13,305 (73.06)	551 (3.03)	
Separated	619 (3.40)	48 (0.26)	
Divorced	1658 (9.10)	189 (1.04)	
**NUMMHS**			
1	10,413 (57.18)	553 (3.04)	0.0015
2	5433 (29.83)	218 (1.20)	
3	1510 (8.29)	84 (0.46)	

In bivariate analyses, discharge status varied by SAP status (*p* < 0.0001): Among clients not having SAP, 14,740 (80.94) were discharged to a private residence and 618 (3.39) to a non‐private residence, whereas 2616 (14.36) and 237 (1.30), respectively, were discharged to a private residence and non‐private residence, respectively.

The majority of the clients were categorized as having SMISED, with 16,369 (89.89%) discharged to a private residence and 825 (4.53%) discharged to a non‐private residence, whereas SMISED was absent in a smaller subgroup, with 987 (5.42%) discharged to a private residence and 30 (0.16%) discharged to a non‐private residence. Overall, residential status at discharge was significantly different by SMIED status (*p* = 0.007) with clients with SMIED showing a greater ratio of non‐private discharge than clients without SMIED. The differences were significant in terms of such key sociodemographic and clinical variables as sex (*p* < 0.0001), age group (*p* < 0.0001), race (*p* = 0.0006), education (*p* < 0.0001), employment status (*p* < 0.0001), marital status (*p* < 0.0001), and NUMMHS (*p* = 0.0015). Ethnicity, on the other hand, did not have significant relationships with discharge setting (*p* = 0.073).

In univariate (unadjusted) models, SAP and SMISED were both found to be related to increased odds of being discharged in a non‐private residence. In particular, 2.16‐fold increased odds were found to be linked to the use of SAP (aOR = 2.16; 95% CI: 1.85–2.52), and 1.66‐fold increased odds were found to be linked to the use of SMISED (aOR = 1.666; 95% CI: 1.14–2.40).

In the adjusted multivariable model, both primary exposures were found to be statistically significant after the adjustment of the sociodemographic and other covariates. SAP was also linked to 40% increased odds of non‐private discharge (aOR = 1.40, 95% CI: 1.18–165), whereas SMISED had about two‐fold increased odds (aOR = 2.10, 95% CI: 1.44–3.05).

Among other covariates, female was associated with 40% lower odds of non‐private discharge (aOR = 0.60, 95% CI: 0.51–0.69) than male. Compared to age groups 0–17, older age groups had significantly higher odds of non‐private discharge (18–44: aOR = 4.97, 95% CI: 3.94–6.28; 45+: aOR = 6.22, 95% CI: 4.77–8.12).

Employment status showed a strong association: unemployed clients had 3.00‐fold higher adjusted odds of non‐private discharge compared with employed clients (95% CI: 2.22–4.04).

Marital status was also significantly associated with non‐private discharge, with 131% increased odds of unmarried (aOR = 2.31, 95% CI: 1.74–3.05), 96% increased odds of separated (aOR = 1.96, 95% CI: 1.33–2.88), and 164% increased odds of divorced (aOR = 2.64, 95% CI: 1.97–3.54) clients compared to married clients.

NUMMHS (number of mental health diagnoses reported for each client) was categorically differentially associated. Adjusted odds of non‐private discharge were 27% lower in clients with two diagnoses (aOR = 0.73, 95% CI: 0.621–0.86) compared to one diagnosis, and no significant difference was found between clients with three diagnoses and the reference group (aOR = 1.18, 95% CI: 0.92–1.5.0) (Table 2).

**TABLE 2:** Univariate and adjusted multivariable logistic regression analysis of the association between relevant covariates and residential status.

Variables	Univariate analysis	Multivariable analysis
	OR (95% CI)	OR (95% CI)
**Primary exposures**		
**Substance abuse problem (SAP)**		
No	Reference	Reference
Yes	2.16 (1.85–2.52)	1.40 (1.18–1.65)
**Serious mental illness and emotional distress (SMISED)**		
No	Reference	Reference
Yes	1.66 (1.14–2.40)	2.10 (1.44–3.05)
**Covariates**		
**Sex**		
Male	Reference	Reference
Female	0.94 (0.82–1.10)	0.60 (0.51–0.69)
**Age**		
0–17	Reference	Reference
18–44	3.18 (2.68–3.78)	4.97 (3.94–6.28)
45+	4.33 (3.62–5.18)	6.22 (4.77–8.12)
**Race**		
Non‐Hispanic Black	Reference	Reference
Non‐Hispanic White	1.20 (1.03–1.40)	0.78 (0.57–1.08)
Non‐Hispanic Other	0.73 (0.54–1.00)	0.98 (0.84–1.15)
**Ethnicity**		
Hispanic	Reference	Reference
Non‐Hispanic	0.73 (0.52–1.03)	0.91 (0.64–1.30)
**Education**		
Less than high school	Reference	Reference
Special education	2.27 (1.98–2.61)	1.397 (1.324–1.473)
High school attendees or graduate	4.22 (2.10–8.55)	1.420 (1.350–1.492)
**Employment status**		
Employed	Reference	Reference
Unemployed	1.80 (1.35–2.40)	3.00 (2.22–4.04)
**Marital status**		
Married	Reference	Reference
Unmarried	1.10 (0.85–1.42)	2.31 (1.74–3.05)
Separated	2.05 (1.40–3.01)	1.96 (1.33–2.88)
Divorced	3.02 (2.27–4.02)	2.64 (1.97–3.54)
**NUMMHS**		
1	Reference	Reference
2	0.76 (0.64–0.89)	0.73 (0.62–0.86)
3	1.05 (0.83–1.33)	1.18 (0.92–1.50)

Abbreviation: CI, confidence interval.

### 3.1 Stepwise Regression Model Using Backward Elimination

Stepwise regression analysis with backward elimination procedure was conducted to identify the most parsimonious model while minimizing issues related to multicollinearity. Starting with the full model with all predictor variables, the backward elimination method systematically removed variables that did not contribute significantly to the model (*p* > 0.05). Variables were sequentially removed on the basis of the statistical significance, with the process continuing until only variables meeting the significance threshold of *α* = 0.05 remained in the final reduced model.

As shown in Table 3, the final model retained the following predictors: Sex, SAP, SMISED, employment status, age group, number of mental health conditions (NUMMHS), and marital status. Several correlated socioeconomic variables that were included in the model initially such as education, race, and ethnicity were removed during the backward elimination process, suggesting that they did not significantly contribute to the model after accounting for other variables. The systematic removal of these correlated attributes suggests that problematic multicollinearity was effectively handled through this variable selection procedure.

**TABLE 3:** Stepwise logistic regression analysis using backward elimination.

Variables	OR (95% CI)	*p* value
**Primary exposures**		
**Substance abuse problem (SAP)**		
No	Reference	0.0002
Yes	1.37 (1.16–1.62)	
**Serious mental illness and emotional distress (SMISED)**		
No	Reference	<0.0001
Yes	2.13 (1.47–3.10)	
**Covariates**		
**Sex**		
Male	Reference	<0.0001
Female	0.59 (0.51–0.68)	
**Age**		
0–17	Reference	<0.0001
18–44	4.95 (4.06–6.01)	
45+	6.20 (4.92–7.81)	
**Employment status**		
Employed	Reference	<0.0001
Unemployed	3.06 (2.27–4.12)	
**Marital status**		
Married	Reference	<0.0001
Unmarried	2.32 (1.76–3.06)	
Separated	1.96 (1.33–2.88)	
Divorced	2.64 (1.97–3.53)	
**NUMMHS**		
1	Reference	0.0003
2	0.74 (0.63–0.87)	
3	1.18 (0.93–1.50)	

Abbreviation: CI, confidence interval.

### 3.2 Moderation of the Employment–Discharge Association by SAP and SMISED Across Race and Ethnicity

Table 4 illustrates the moderating effect of SAP and SMISED on the employment status, stratified by race and ethnicity for predicting the residential status at discharge.

**TABLE 4:** Moderating effect of substance abuse problems (SAP) and serious mental illness/serious emotional distress (SMISED) on employment status across race and ethnicity.

Employment status *(SAP, SMISED)	White	Black	Hispanic/Latino	Non‐Hispanic/Latino	Others
	Odds ratio (95% CI)	Odds ratio (95% CI)	Odds ratio (95% CI)	Odds ratio (95% CI)	Odds ratio (95% CI)
Unemployed vs. employed (reference) when “SAP = Yes”	1.51*** (1.23–1.86)	1.85** (1.29–2.66)	3.74** (1.65–8.46)	1.57*** (1.32–1.87)	2.33* (1.28–4.34)
Unemployed vs. employed (reference) when “SMISED = Yes”	2.93*** (2.05–4.20)	4.02*** (2.00–8.37)	1.06 (0.40–2.90)	3.10*** (2.38–4.01)	8.66* (1.13–66.50)

*Note:* Asterisks indicate levels of statistical significance; all analyses were controlled for other covariates; “SAP = No” and “SMISED = No” were used as the reference categories and served as the baselines for each case.

Abbreviation: CI, confidence interval.

*p < 0.05.

**p < 0.005.

***p < 0.0005.

**When SAP = Yes**: For all racial and ethnic groups, unemployed clients had significantly higher odds of non‐private discharge compared to employed clients. White clients had 51% higher odds (aOR = 1.51, 95% CI: 1.23–1.86), Black clients had 85% higher odds (aOR = 1.85, 95% CI: 1.29–2.66), non‐Hispanic/Latino clients had 57% higher odds (aOR = 1.57, 95% CI: 1.32–1.87), and “Others” had more than double the odds (aOR = 2.33, 95% CI: 1.28–4.34). Hispanic/Latino clients showed the highest association (aOR = 3.74, 95% CI: 1.65–8.46) which indicated that individuals with the SAP, unemployment was associated with significantly higher odds of non‐private discharge among Hispanic/Latino clients compared to employed Hispanic/Latino clients.

**When SMISED = Yes**: Unemployed White clients had nearly three‐fold higher odds (aOR = 2.93, 95% CI: 2.05–4.20), and unemployed Black clients had approximately four‐fold higher odds (aOR = 4.02, 95% CI: 2.00–8.37) of non‐private discharge, compared with employed clients within the same race group. Likewise, unemployment was also linked to nearly triple the odds (aOR = 3.10, 95% CI: 2.3–84.01) among non‐Hispanic/Latino clients. Conversely, unemployment was not significantly related to non‐private discharge among Hispanic/Latino clients with SMISED (aOR = 1.06, 95% CI: 0.40–2.90).

### 3.3 Model Diagnostics

To assess the overall model fit, discriminative ability, and the role of individual observations, model diagnostics were performed using deviance residuals, receiver operating characteristic (ROC) curve, and deletion Pearson chi‐square statistics (DIFCHISQ). The plotted deviance residuals (Figure S1) when plotted against predicted probabilities had the typical two band structure of binary logistic regression that reflects the observed non‐private and private residential outcomes. The residuals were symmetrically distributed around zero with no clear systematic patterns throughout the scale of predicted probabilities and showed that the model functional form fitted the analysis well and that there was no sign of large lack of fit or extreme outliers.

The ROC curve (Figure S2) was used to test the model discrimination.

The area under the curve (AUC) was 0.72, which suggested that model was capable of discriminating between “individuals being discharged to non‐private residence” and “those discharged to private residence” across a series of classification thresholds. The leverage values and deletion Pearson chi‐square statistics (DIFCHISQ) (Figure S3) were used to test influence diagnostics. Although few observations had high values of DIFCHISQ, high values of leverage were not observed, and this indicated that such points did not influence the results of the estimated regression coefficients. All figures are added to the Supporting Information file.

## 4 Discussion

This study investigated the relationship between residential status and behavioral or cognitive disorders in the United States, using the 2022 MH‐CLD [6]. The results demonstrated that those with behavioral or cognitive disorders are significantly less likely to reside in private residences compared to experiencing homelessness or living in alternative settings, such as foster care or correctional facilities. Covariates such as education, gender, employment status, and socioeconomic factors were found to significantly influence residential outcomes, with higher education and employment levels improving the likelihood of residential stability. These findings align with prior literature emphasizing the vulnerabilities of those with mental health conditions in institutional and unstable living arrangements [2, 9], while providing new insights into the specific demographic and socioeconomic predictors that affect residential outcomes. The interpretation of these findings highlights the broader impact of socioeconomic disparities. For instance, the strong relationship between higher education and private residence stability underscores the importance of access to resources, whereas the higher prevalence of homelessness among children with disorders emphasizes gaps in social safety nets [13]. Gender differences in outcomes, where females are less likely to experience homelessness than males, might reflect differential access to social support services or cultural dynamics [1, 8, 9, 15]. Additionally, the partial mediation of these outcomes by socioeconomic factors indicates complex mechanisms involving support, mental health literacy, and systemic inequalities [2, 3].

Substance use emerged as a significant factor influencing residential stability, with those experiencing substance use problems being 20.2% less likely to reside in private residences compared to those without such issues. This shows the widespread impact of substance use on housing outcomes across populations, reflecting both individual‐level challenges and broader systemic barriers. Substance use often co‐occurs with other mental health conditions, creating a cycle of instability that limits access to secure and supportive housing. Addressing these issues through comprehensive intervention strategies, including integrated mental health, substance use treatment, and housing support programs, is important when trying to improve residential outcomes and overall well‐being for affected individuals.

The findings from the stratified moderation analysis by race and ethnicity indicate that unemployment operates as a significant socioeconomic “risk amplifier” for adverse post‐discharge residential status when co‐occurring with SAP and/or SMISED, with increased odds observed across most racial and ethnic groups. Conceptually, this trend is consistent with a multidimensional model in which clinical burden (SAP and SMISED) compromises functional capacity and continuity of care, whereas unemployment constrains income and housing access, together increasing vulnerability to housing instability during the discharge‐to‐community transition. These findings are also consistent with structural evidence, which shows that labor and housing market factors, safety‐net resources, and discriminatory obstacles differentially determine the risk of homelessness among racial groups, such that the summed clinical and socioeconomic cost might have a stronger impact on a specific group than the rest [25].

Besides the social and behavioral pathways discussed, there is also a neurobiological explanation to our findings that can be used to explain why some individuals would be more functionally impaired and have a higher residential instability after discharge. Although the MH‐CLD data are administrative and cross‐sectional and therefore cannot test mechanistic pathways, converging neurobiological models suggest that impairments in neural systems supporting self‐regulation, cognitive control, salience processing, and stress responsivity may plausibly translate into functional difficulties relevant to the post‐discharge transition, including planning, routine maintenance, conflict negotiation, and sustained engagement with outpatient care and housing‐related demands. Recent syntheses emphasize that variation in prefrontal and large‐scale network functioning is linked to adaptive coping and real‐world functioning in the context of psychiatric risk, offering a complementary, mechanism‐oriented framing for why some individuals may experience greater functional impairment and residential instability after discharge [23]. Neurocomputational views, which emphasize the role of self‐representation and multiscale control processes, can potentially offer a valuable conceptual linkage between the capacity of individual‐level regulation and more complex, high‐stakes decisions, which are made during the discharge‐to‐community transition (e.g., treatment adherence, help‐seeking, and social decision‐making) [24].

The practical implications of our findings facilitate the design of specific interventions aimed at the high‐risk transition between living in the facilities and living in the community. Clients with comorbid SMISED and substance abuse issues can use housing‐first or permanent supportive housing (PSH) practices that disconnect housing access with symptom remission or abstinence and combine rent subsidies with ongoing services, which is consistently related to better housing stability in individuals with severe mental illness [22]. Moreover, as discharge is a predictable high‐risk period of residential disruption, interventions like CTI provide a feasible model: time‐limited, intensive case management plus inpatient‐community transitions, enhancement of linkages to outpatient care and benefits, and the active planning of housing are proven to lower post‐discharge homelessness [21].

In the post‐pandemic context, these findings are particularly salient. Recent large‐scale studies and federal surveillance reports document sharp increases in housing insecurity among individuals with psychiatric conditions, driven by rising housing costs, constrained affordable housing supply, and growing demand for community‐based mental health services [9, 13]. In response, the US Department of Housing and Urban Development (HUD) has expanded initiatives emphasizing Housing First and PSH models as core strategies for stabilizing individuals with SMI. Recent surveys of the US homelessness epidemiology keep reporting high rates of co‐occurring SMI and substance use disorder in adults experiencing homelessness, so the identified subgroups are at increased risk of unstable housing during the time of discharge are practical [18]. In line with post‐pandemic evidence of an increasing burden of residential insecurity in the United States and its strong overlap with behavioral health demands, our findings explores the role of associated cognitive, behavioral, and sociodemographic factors on the residential instability, among a national mental health treatment dataset, [12].

The present study contributes timely, nationally representative evidence from the MH‐CLD system, which remains the most recent standardized client‐level dataset from state mental health authorities. As such, our findings complement emerging post‐pandemic housing and mental health initiatives and provide an empirical foundation for ongoing federal and state reform efforts.

Although this study contributes to valuable insights, several limitations should be noted. The cross‐sectional design limits causal inferences, as associations are captured at a single point in time [4]. The reliance on categorized and self‐reported data may introduce inaccuracies in diagnostic and residential classifications [6]. Furthermore, the exclusion of secondary and tertiary diagnoses may underestimate the broader mental health burden, and any missing data may slightly affect the robustness of findings. Moreover, the absence of longitudinal follow‐up limits the understanding of transitions in residential stability over time, a point noted as a gap in prior literature as well [2]. Furthermore, the analytic sample also represents providers and facilities that are reported to the MH‐CLD system at SAMHSA; therefore, service settings with incomplete reporting or non‐reporting at all (some community‐based or private providers) might be underrepresented, which may impact generalizability. In addition, the analyses were limited to complete cases, and therefore, systematic differences between the excluded and retained records could have introduced selection bias. Finally, stepwise procedure may be vulnerable to sample‐specific variability and can yield unstable variable selection with potentially optimistic effect estimates; accordingly, the retained predictors should be viewed as providing a parsimonious empirical model for this dataset rather than definitive causal determinants or universally generalizable predictors.

Future research should employ longitudinal designs to capture the dynamic relationship between mental health and residential outcomes, while also addressing unmeasured variables such as cultural influences and systemic barriers to care [4, 9]. Moreover, the integration of quantitative data with client lived experiences through mixed‐methods approaches can allow researchers to develop a complete understanding of stable housing barriers and facilitators which leads to the creation of culturally sensitive patient‐centered interventions.

These findings suggest important implications for public health and policy. Interventions should focus on addressing socioeconomic barriers, such as caregiver education and employment, to improve residential stability for children with mental health disorders [9, 13]. Housing programs integrated with mental health services could provide holistic support, addressing both immediate and underlying needs. The policy approach needs to advance past clinical treatment by creating supportive housing programs that unite stable housing with behavioral health care services and by improving financial and housing assistance access and by reducing racial and ethnic minority group and low‐resource community disparities. The reduction of administrative barriers and the achievement of equitable access and sustainable recovery depend on cross‐sector collaboration between housing authorities and healthcare providers and social service agencies. Researchers should implement policies that address both clinical and socioeconomic determinants of housing stability to enhance treatment adherence and reduce relapse and hospitalizations which will lead to better long‐term outcomes for this vulnerable population.

## 5 Conclusion

This study highlights the significant associations between behavioral and cognitive disorders and residential status amongst different age groups, emphasizing the role of socioeconomic and demographic factors in shaping housing outcomes. The findings underscore the urgent need for targeted interventions that address housing stability and its interplay with mental health, particularly among vulnerable populations, like those dealing with substance use problems. By shedding light on these critical associations, this research provides a foundation for future studies and practical interventions aimed at reducing disparities and improving residential outcomes for all with mental health conditions.

## Author Contributions

All authors contributed equally to the conception and design of the study, data analysis and interpretation, and manuscript preparation. Each author participated in conceptualization, methodology, formal analysis, data curation, writing – original draft, and writing – review and editing. All authors approved the final version of the manuscript and agree to be accountable for all aspects of the work.

## Ethics Statement

This observational study used de‐identified, publicly available data from the Substance Abuse and Mental Health Services Administration (https://www.samhsa.gov/data/report/2022‐mental‐health‐client‐level‐data‐annual‐report), which contain no personal identifiers or protected health information. As such, the study did not involve human subjects as defined by 45 CFR 46, and institutional review board (IRB) approval was not required. The study followed the ethical standards outlined in the Declaration of Helsinki and adheres to the STROBE guidelines for reporting observational studies.

## Consent

The authors have nothing to report.

## Conflicts of Interest

The authors declare no conflicts of interest.

## Supporting information




**Supporting File 1:** puh270218‐sup‐0001‐SuppMat.docx.

## Data Availability

The data that support the findings of this study are available from the corresponding author upon reasonable request.
