# Supplementary material for: The Impact of Substance Abuse Problems and Serious Mental Illness/Serious Emotional Distress on Post‐Discharge Residential Status Among Clients With Behavioral and Cognitive Disorders: Evidence From SAMHSA MH‐CLD Data
Source: Public Health Chall. 2026 Apr 10;5(2):e70218. doi: 10.1002/puh2.70218 (PMC13067978; doi:10.1002/puh2.70218)

**Model Diagnostics Plots**

**Supplementary Figure 1: The Deviance Residuals vs. Predicted Probabilities Plot**


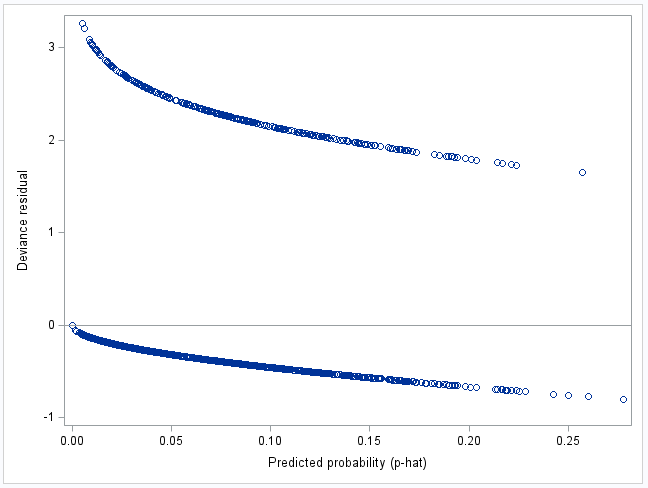


**Supplementary Figure 2: AUC-ROC Curve Showing Predictive Accuracy**


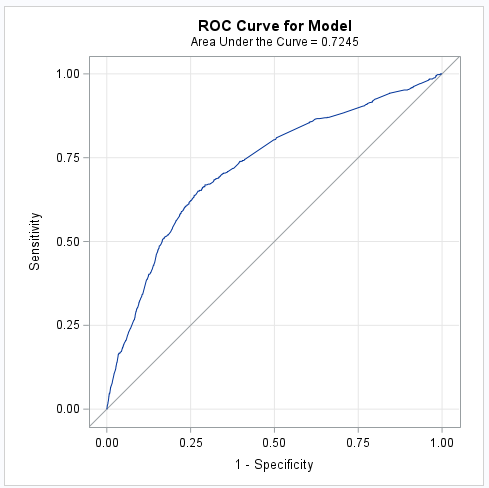


**Supplementary Figure 3: Deletion Pearson chi-square (DIFCHISQ) vs. Leverage Plot**


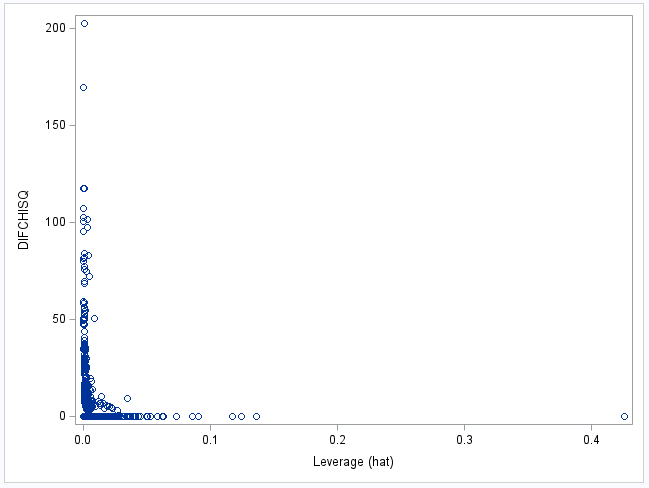

Supplement: Supplementary file 1 — Supporting File 1: puh270218‐sup‐0001‐SuppMat.docx. [file PUH2-5-e70218-s001.docx]
